# Effect of multivitamin drug on intractable dry eye symptoms

**DOI:** 10.3389/fmed.2022.978107

**Published:** 2022-09-06

**Authors:** Se Hie Park, Jin Sun Hwang, Young Joo Shin

**Affiliations:** ^1^Department of Ophthalmology, Hallym University Medical Center, Hallym University College of Medicine, Seoul, South Korea; ^2^Hallym BioEyeTech Research Center, Hallym University College of Medicine, Seoul, South Korea

**Keywords:** dry eye symptoms, dryness, intractable dry eye, multivitamin drug, nutrition

## Abstract

Dry eye is a disorder of tear film and ocular surface characterized by ocular discomforts. It is associated with multiple causes and sometimes intractable. We investigated the effect of oral multivitamin supplementation (MVG) on dry eyes. Tear break-up time (TBUT), fluorescein ocular surface staining score, and tear secretion Schirmer test were measured in dry eye patients refractory to conventional topical treatment. The ocular surface disease index (OSDI), visual analog pain score (VAS), and modified standardized patient evaluation of eye dryness questionnaire were analyzed. In total, 42 eyes of 42 patients were included. TBUT increased at 1 and 3 months compared to baseline (*p* < 0.05). OSDI decreased at 1 and 3 months compared to baseline (*p* < 0.05). VAS score, impact on life, and frequency of total symptoms decreased at 3 months compared to baseline (*p* < 0.05). Oral administration of MVG, a vitamin complex formulation, was effective in stabilizing tear stability and alleviating symptoms in patients with intractable dry eye. Thus, it may be a viable treatment option for intractable dry eye.

## Introduction

Dry eye is an ocular surface disease that is characterized by symptoms including irritation and discomfort of the ocular surface caused by abnormal tear film and ocular surface inflammation (1). Tear film contains IgA, lysozyme, lactoferrin, lipocalin, phosphatidylcholine, phosphatidylethanolamine, cholesterol oleate, triglycerides, and free fatty acids (2). The composition of tears changes according to factors such as age, systemic diseases, and hormones (3). Dry eye is commonly treated with artificial tears, topical secretagogues, immunomodulators, or punctal occlusion (1). Although the treatment for dry eye syndrome is usually local, the risk factors for dry eye syndrome are generally systemic. Risk factors for dry eye syndrome include hormonal changes, aging, diabetes, thyroid disease, tobacco use, lack of sleep, depression, and drug use (3). It has been reported that vitamin D, vitamin A, vitamin B12, and antioxidants may be helpful as nutritional supplements related to dry eye (4). A lot of research has been performed for the treatment of dry eye, but the intractable dry eye, not responding to topical treatment, remains challenging (1, 5, 6). This may be due to a decrease in tear film quality resulting from changes in the composition of basally secreted tears or molecular changes in the ocular surface (7). In addition, nutritional deficiency may be one of the causes of intractable dry eye.

Oxidative stress has been reported as one of the mechanisms in dry eyes (8, 9). Oxidative stress is generated as a byproduct when energy is produced in mitochondria and normally plays a physiologic role in cell signaling (10). However, excessive oxidative stress damages DNA, RNA, or cell proteins, causing cell death and inducing inflammation although tears and ocular surfaces have antioxidant systems (11, 12). Oxidative stress increases with age and contributes to neuropathy and excitability (13). Resolving oxidative stress and neuropathy by oral administration of antioxidants or vitamins may improve ocular symptoms. Vitamins reduce dry eye symptoms. Tocopherol, ascorbic acid, and β-carotene are antioxidants that reduce oxidative stress and inflammation of the ocular surface (14). Thiamine nitrate (15) and pyridoxine (14) have been reported to ameliorate dry eye. Oral multivitamin drug (MVG; Greenwol soft cap, Kyungnam Pharm.) has been developed to reduce dry eye symptoms and includes tocopherol, ascorbic acid, thiamine, riboflavin, pyridoxine, β-carotene, zinc oxide, selenium, and ubidecarenone. In this study, we investigated the protective effect of MVG in intractable dry eyes.

## Materials and methods

### Patients

This prospective study was approved by the Hallym University Medical Center Institutional Review Board and was carried out following the principles of the Declaration of Helsinki between January 2018 and May 2021, and patients with dry eye symptoms refractory to topical treatment were prescribed the oral administration of MVG. Informed consent was obtained from all patients involved in this study. Patients who met the following criteria were included in the study: older than 45 years of age; presence of clinical signs and symptoms of dry eye; presence of symptoms refractory to treatment with artificial tears, bioactive drugs, and punctal plug; and clinical examination variable assessments and survey completions within 1 month and/or 3 months after prescribing oral MVG. Topical treatment was continuously prescribed. MVG includes tocopherol acetate 200 mg, ascorbic acid 450 mg, thiamine nitrate 12.5 mg, riboflavin 6 mg, pyridoxine HCl 25 mg, β-carotene 30% suspension 10 mg, zinc oxide 17.43 mg, selenium 0.1% powder 25 mg, and ubidecarenone 5 mg. Oral MVG was prescribed twice daily.

### Ophthalmic examination

The survey was conducted using the ocular surface disease index (OSDI), visual analog pain scale (VAS), and a modified standardized patient evaluation of eye dryness (SPEED) questionnaire before and after 1 and 3 months of MVG administration. Tear break-up time (TBUT), fluorescein staining score (FSS), eyelid margin hyperemia, and tear secretion were measured. OSDI was calculated after patient OSDI questionnaire completion for measuring dry eye symptoms severity (16). OSDI is scored on a scale of 0–100 with greater scores representing greater disability (16). VAS, which measured ocular pain intensity, was assessed using a segmental numerical technique (17), where 0 = no discomfort and 10 = maximal discomfort (18). A modified SPEED questionnaire was used to assess the frequency and severity of each symptom, including dryness, foreign body sensation (FBS), coldness, eye fatigue, pain, and photophobia (19). The SPEED questionnaire was scored for both frequency of symptoms (0 = no symptoms, 1 = sometimes, 2 = often, and 3 = constant) and severity of symptom/impact on daily life (0 = no symptoms, 1 = tolerable but not uncomfortable, 2 = uncomfortable but does not interfere with my day, 3 = bothersome and interferes with my day, and 4 = intolerable and unable to perform my daily tasks).

Tear break-up time, which indicates tear stability, was measured using fluorescein staining strips (Haag-Streit Strips, Haag-Streit, United States) and slit lamp microscopy without stimulation (18). Briefly, a drop of normal saline was instilled to the strip, and excessive liquid was removed by shaking strip. Then, the strip was touched to inferior conjunctiva. After blinking several times, the time between the last blinking and the first dry spot was measured as TBUT (20). Longer TBUT represents the stable tear film and shorter TBUT is considered as the unstable tear film (21). FSS was assessed after TBUT test under yellow filter and graded using the Oxford grading scale (22). FSS is defined as the degree of punctate keratitis and higher score is considered as more severe FSS (23). Eyelid margin hyperemia was graded using slit lamp microscopy. Eyelid margin hyperemia indicates the inflammation of eyelid and higher score represents the inflammation of eyelid (20). Tear secretion was measured using the Schirmer strip test without anesthesia. A fine strip paper was placed inside the lower conjunctival sac and the wet part was measured (20).

### Statistics

Data are expressed as the mean ± standard deviation. Statistical analyses were performed using unpaired Student’s *t*-test for two-group comparisons and one-way analysis of variance, followed by Tukey’s multiple comparison test for more than two groups using GraphPad Prism v.9 (GraphPad Software, San Diego, CA, United States).

## Results

In total, 42 eyes of 42 patients were included in the study. Seven (16.7%) men and 35 (83.3%) women, with a mean age of 53.64 ± 12.06 years were enrolled. Forty-two (100%) patients returned 1 and 3 months after prescribing oral MVG. At baseline, TBUT was 4.61 ± 2.10 s, FSS was 0.59 ± 0.92, and lid hyperemia was 1.59 ± 1.00. OSDI and VAS were 48.13 ± 22.78 and 3.17 ± 2.77, respectively. Impact on the day life and daily frequency were 2.62 ± 0.80 and 2.29 ± 1.04.

**FIGURE 1:**
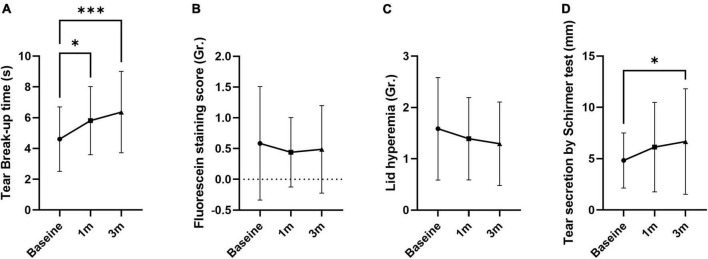
The effect of MVG on objective signs of dry eye. **(A)** Tear break-up time was improved. **(B,C)** Fluorescein staining score and lid hyperemia were not changed. **(D)** Tear secretion by Schirmer test was improved. Data presented as mean ± SD. *p < 0.05, **p < 0.01, and ***p < 0.001.

**FIGURE 2:**
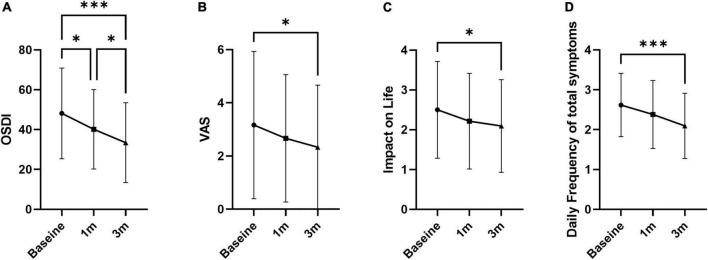
Overall symptoms of dry eye. **(A)** Ocular surface disease index. **(B)** Visual analog pain score. **(C)** Impact of symptoms on daily life. **(D)** Daily frequency of total symptoms. Data presented as mean ± SD. *p < 0.05, **p < 0.01, and ***p < 0.001.

Tear break-up time increased at 1 and 3 months compared to baseline by 26.0 and 38.2%, respectively (5.81 ± 2.21 s at 1 month and 6.37 ± 2.64 at 3 months; *p* = 0.018 and 0.001; Figure 1A and Table 1). FSS and lid hyperemia did not change (Figures 1B,C). Tear secretion increased at 3 months compared to baseline (4.95 ± 2.73 mm at baseline vs. 6.78 ± 5.24 mm at 3 months; *p* = 0.030; Figure 1D). OSDI decreased at 1 and 3 months compared to baseline by 16.6 and 30.5% (40.16 ± 19.93 at 1 month and 33.44 ± 19.93 at 3 months; *p* = 0.011 and *p* < 0.001; Figure 2A). VAS, impact on life, and frequency of total symptoms were decreased at 3 months compared to baseline by 20.1, 19.8, and 18.8% (2.31 ± 2.31, 2.10 ± 0.82, and 1.86 ± 0.95; *p* = 0.029, 0.042, and <0.001; Figures 2B–D).

**TABLE 1:** Signs and general symptoms change before and after taking oral MVG.

	Before taking oral MVG	One month after oral MVG	Three months after oral MVG
*N*	42	42	42
Male:female	7:35	7:35	7:35
TBUT (s)	4.61 ± 2.10	5.81 ± 2.21	6.37 ± 2.64
FSS	0.59 ± 0.92	0.44 ± 0.57	0.46 ± 0.70
Lid hyperemia	1.59 ± 1.00	1.39 ± 0.80	1.29 ± 0.81
OSDI	48.13 ± 22.78	40.16 ± 19.93	33.44 ± 19.93
VAS	3.17 ± 2.77	2.67 ± 2.40	2.31 ± 2.31
Impact on the daily life	2.62 ± 0.80	2.38 ± 0.85	2.10 ± 0.82
Daily frequency	2.29 ± 1.04	2.07 ± 1.07	1.86 ± 0.95

Data are expressed as mean ± SD.

**FIGURE 3:**
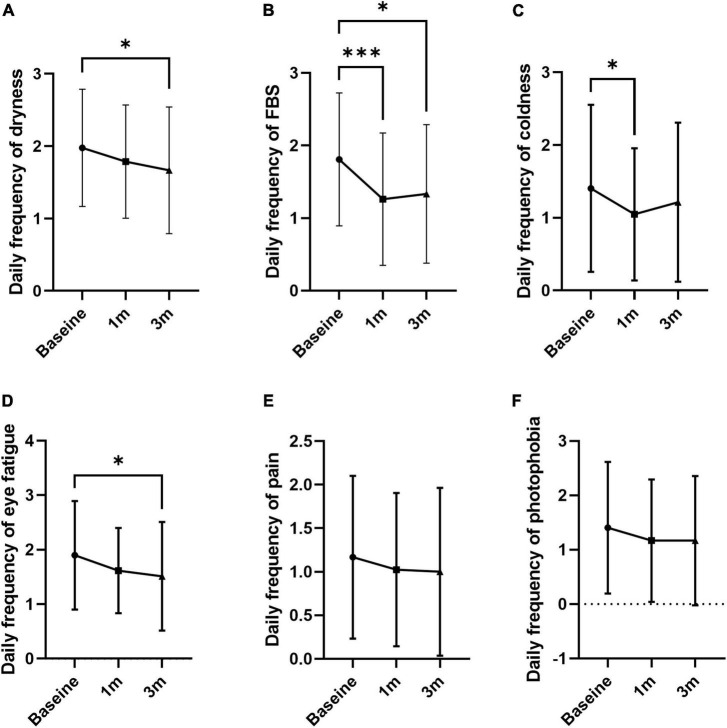
The effect of MVG on the frequency of each dry eye symptom. **(A)** Dryness. **(B)** Foreign body sensation. **(C)** Coldness. **(D)** Eye fatigue. **(E)** Pain. **(F)** Photophobia. * < 0.05, ** < 0.01, and ***p < 0.001.

**FIGURE 4:**
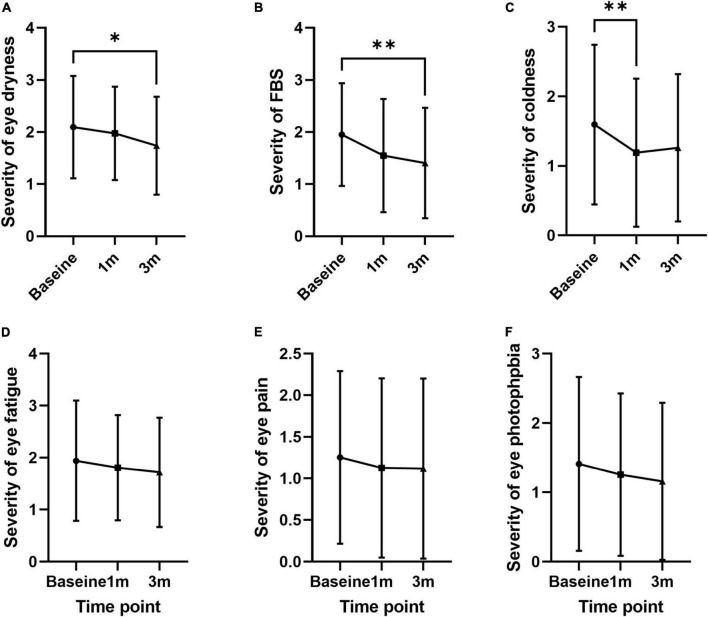
The effect of MVG on the severity of each dry eye symptom. **(A)** Dryness. **(B)** Foreign body sensation. **(C)** Coldness. **(D)** Eye fatigue. **(E)** Pain. **(F)** Photophobia. * < 0.05 and ** < 0.01.

The frequency of individual symptoms was evaluated (Table 2). Dryness frequency decreased at 3 months compared to baseline by 12.7% (1.67 ± 0.87; *p* = 0.049; Figure 3A). FBS frequency decreased at 1 and 3 months compared to baseline by 30.4 and 26.5% (1.26 ± 0.91 and 1.33 ± 0.95; *p* = 0.005 and 0.012, respectively; Figure 3B). Coldness frequency decreased at 1 month compared to baseline by 25.0% (1.05 ± 0.91; *p* = 0.011; Figure 3C). The frequency of eye fatigue, pain, and photophobia did not change after oral MVG administration (Figures 3D–F). The severity of individual symptoms was also evaluated. The severity of dryness and FBS decreased at 3 months compared to baseline by 17.1 and 28.2% (1.40 ± 1.06; *p* = 0.049 and 0.004; Figures 4A,B). Coldness severity decreased at 1 month compared to baseline by 25.6% (1.19 ± 1.07; *p* = 0.005; Figure 4C). The frequency of eye fatigue, pain, and photophobia did not change after oral MVG administration (Figures 4D–F).

**TABLE 2:** Changes of frequency and severity of each symptom before and after taking oral MVG.

	Before taking oral MVG	One month after oral MVG	Three months after oral MVG
**Frequency**			
Dryness	1.98 ± 0.81	1.79 ± 0.78	1.67 ± 0.87
FB sensation	1.81 ± 0.92	1.26 ± 0.91	1.33 ± 0.95
Coldness	1.40 ± 1.15	1.05 ± 0.91	1.21 ± 1.10
Fatigue	1.86 ± 1.03	1.62 ± 0.76	1.48 ± 0.99
Pain	1.17 ± 0.94	1.02 ± 0.88	1.00 ± 0.96
Photophobia	1.40 ± 1.21	1.17 ± 1.12	1.17 ± 1.19
**Severity**			
Dryness	2.10 ± 0.98	1.98 ± 0.90	1.74 ± 0.94
FB sensation	1.95 ± 0.99	1.55 ± 1.09	1.40 ± 1.06
Coldness	1.60 ± 1.15	1.19 ± 1.07	1.26 ± 1.06
Fatigue	1.93 ± 1.11	1.76 ± 0.98	1.64 ± 1.08
Pain	1.31 ± 1.05	1.14 ± 1.05	1.14 ± 1.05
Photophobia	1.43 ± 1.41	1.24 ± 1.17	1.14 ± 1.14

Data are expressed as mean ± SD.

## Discussion

Dry eye is a disease of the tear film and ocular surface affected by systemic nutritional status (4, 24). In this study, we investigated the effect of MVG on dry eye symptoms. The main mechanism of dry eye involves oxidative stress and inflammation (8, 25–27). Basic fibroblast growth factor (b-FGF) and L-cysteine supplementation has been suggested to promote the corneal epithelial healing and reduce the corneal haze (26–28). Oxidative stress causes inflammation through activation of nuclear factor-kappa B (NF-κB) signaling and secretion of senescence-associated secretary phenotypes (29). The imbalance between ROS and endogenous antioxidative system could result in neuropathic pain (30, 31). Neuropathic pain can be alleviated by inhibiting oxidative stress-induced NF-κB activation or by blocking protein kinase C (31, 32). Thus, the reduction of oxidative stress is necessary for minimizing inflammation and managing dry eye. The MVG used in this study consists of a vitamin supplement with antioxidant action that helps in dry eye syndrome (33). MVG includes tocopherol (vitamin E), ascorbic acid, thiamine (vitamin B1), riboflavin (vitamin B2), pyridoxine (vitamin B6), β-carotene (vitamin A precursor), zinc oxide, selenium, and ubidecarenone (coenzyme Q10). Oral administration of MVG improved the symptoms and signs of dry eye in this study.

Intractable dry eyes may be due to neuropathic pain or nutrition deficiency (34). Oxidative stress is critically involved in neuropathic pain and contributes to exaggeration of pain hypersensitivity during persistent pain (30, 34). Tocopherol, an antioxidant, has an analgesic effect by reducing central sensitization in neuropathic pain (35) and preventing peripheral neuropathy (36). Topical eyedrops of 0.1% crosslinked hyaluronic acid, coenzyme Q10, and vitamin E improve dry eye (37). Ascorbic acid, one of the antioxidants contained in tear films, protects the ocular surface against the oxidative stress (38). Supplementation of ascorbic acid shows antinociceptive effects (39, 40). It has been reported that the combination of vitamin C and vitamin E attenuates the neuropathic pain behavior by synergistic antinociceptive effects, although vitamin C or vitamin E given alone failed to reduce the nociceptive behavior (39). Thiamine, which reduces peripheral neuropathic pain (41), attenuates symptoms of dry eye (15). Riboflavin supplementation reduces hyperalgesia and inflammation (42) and inhibits histamine-dependent itch by modulating transient receptor potential vanilloid 1 (43). Pyridoxine is used for the management of sicca syndrome (14) and improves neuropathic pain, such as allodynia and hyperalgesia, *via* suppression of oxidative–nitrosative stress, pro-inflammatory cytokines secretion, and apoptosis (44). β-Carotene is a precursor of vitamin A, deficiency of which leads to xerophthalmia and ocular surface inflammation (45, 46). Topical and oral vitamin A supplementation reduces dry eye signs and symptoms, also promoting goblet cell proliferation (47–49). Zinc is a part of many essential enzymes, including superoxide dismutase (50). The absence of zinc or zinc deficiency is associated with apoptosis, DNA damage, and changes immune function, partly due to the important role of zinc in countering oxidative stress (51). Topical zinc-hyaluronate improves ocular surface symptoms by decreasing corneal mechano- and polymodal receptor excitability (52). Selenium protects the ocular surface from oxidative stress by reducing matrix metallopeptidase-9, interleukin-6, and 8-hydroxy-2′-deoxyguanosine (53, 54). Coenzyme Q10 prevents peripheral neuropathy and neuropathic pain and attenuates neuron loss (55, 56). Eyedrops containing crosslinked hyaluronic acid and CoQ10 restore the health of ocular surface (57), and coenzyme Q10 protects lacrimal glands against the oxidative stress (58).

## Conclusion

Conventional treatments often fail to improve dry eye symptoms, which may be associated with oxidative stress or systemic causes. Oral administration of MVG, a vitamin complex formulation, was effective in stabilizing tear stability and alleviating symptoms in patients with intractable dry eye syndrome. Thus, it may be a viable treatment strategy for intractable dry eye.

## Data availability statement

Datasets are available from the corresponding author on reasonable request.

## Ethics statement

This prospective study was approved by the Hallym University Kangnam Sacred Heart Hospital Institutional Review Board. The patients/participants provided their written informed consent to participate in this study.

## Author contributions

Acquisition of data: SP, and YS. Analysis and/or interpretation of data: SP, JH, and YS. Drafting the manuscript: SP, JH, and YS. Revising the manuscript critically for relevant intellectual content: YS. Approval of the version of the manuscript to be published: SP, JH, and YS.
